# End-of-Life Care and Health Care Spending for Medicare Beneficiaries With Dementia in Accountable Care Organizations

**DOI:** 10.1001/jamahealthforum.2025.0731

**Published:** 2025-05-09

**Authors:** Jessica J. Zhang, David B. Reuben, Anne M. Walling, David S. Zingmond, Cheryl L. Damberg, Neil S. Wenger, Haiyong Xu, Ryo Ikesu, Gillian S. Kaneshiro, Alexandra Klomhaus, Hiroshi Gotanda, Yusuke Tsugawa

**Affiliations:** 1 Division of General Internal Medicine and Health Services Research, Department of Medicine, David Geffen School of Medicine at University of California, Los Angeles; 2 National Clinician Scholars Program, University of California, Los Angeles; 3 Multicampus Program in Geriatric Medicine and Gerontology, University of California, Los Angeles; 4 Veterans Affairs Greater Los Angeles Healthcare System, Los Angeles, California; 5 RAND Corporation, Santa Monica, California; 6 Department of Epidemiology, Fielding School of Public Health, University of California, Los Angeles; 7 Division of General Internal Medicine, Cedars-Sinai Medical Center, Los Angeles, California; 8 Department of Health Policy and Management, Fielding School of Public Health, University of California, Los Angeles

## Abstract

**Importance:**

Individuals with dementia may receive high-intensity care at the end of life (EOL) that does not align with their preferences and is costly. Medicare Accountable Care Organizations (ACOs) are an alternative payment model that aims to incentivize high-quality care and lower spending.

**Objective:**

To compare EOL care processes, outcomes, and health care spending between Medicare beneficiaries with dementia in a Medicare Shared Savings Program (MSSP) ACO and non-ACO.

**Design, Setting, and Participants:**

This quasi-experimental study of EOL care used a nationally representative 20% random sample of Medicare fee-for-service beneficiaries with dementia (age ≥66 years) who died from 2017 to 2020. Difference-in-differences and event study design approaches were used to compare outcomes between beneficiaries attributed to MSSP ACO vs those who were not. Data were analyzed from June 2023 to December 2024.

**Exposure:**

MSSP ACO entry from 2017 to 2019 vs non-ACO.

**Main Outcomes and Measures:**

Differential changes in 5 areas: (1) billing for advance care planning; (2) palliative care counseling in last 6 months of life; (3) hospice in last 6 months of life; (4) high-intensity care in last 30 days of life (ie, emergency department visit, hospitalization, intensive care unit admission, in-hospital death, cardiopulmonary resuscitation or mechanical ventilation, feeding tube placement); and (5) health care spending in last 6 months of life.

**Results:**

Of 162 034 eligible Medicare beneficiaries (mean [SD] age, 85.0 [7.9] years; 94 304 female [58.2%]), 51 191 (31.6%) were attributed to MSSP ACO. Adjusted trends in outcomes were similar between ACO and non-ACO groups before ACO entry. The difference-in-differences analyses found no evidence that EOL care processes or outcomes (eg, hospice in last 6 months of life, −0.4 percentage points [pp]; 95% CI, −1.4 pp to 0.5 pp; *P* > .99) or spending (eg, total health care spending in last 6 months of life, −$632; 95% CI, −$1377 to $113; *P* = .96) differed between beneficiaries treated in ACOs vs non-ACOs. The event study design also showed no evidence of differential changes in outcomes between the 2 groups. Sensitivity analyses using inverse probability weighting yielded similar results.

**Conclusions and Relevance:**

Using nationally representative data on beneficiaries with dementia at EOL, this quasi-experimental study found no evidence that EOL care processes, outcomes, or spending changed with ACO entry for Medicare fee-for-service beneficiaries vs non-ACO beneficiaries. Alternative payment models to ACOs may be needed to coordinate high-quality care with lower spending for beneficiaries with dementia at the EOL.

## Key Points

**Question:**

For beneficiaries with dementia, how do end-of-life care processes, outcomes, and health care spending differ between beneficiaries attributed to a Medicare Shared Savings Program Accountable Care Organization (ACO) compared with those who were not?

**Findings:**

This study of 162 034 Medicare fee-for-service beneficiaries who died from 2017 through 2020 found no evidence of differences in end-of-life care processes, outcomes, or spending between beneficiaries in ACO vs non-ACO.

**Meaning:**

These findings suggest that alternative payment models to ACOs may be needed to coordinate high-quality care with lower health care spending for Medicare beneficiaries with dementia at the end of life.

## Teaser

This quasi-experimental study evaluates end-of-life care processes, outcomes, and spending for beneficiaries with dementia who are or a not in a Medicare Accountable Care Organization.

## Introduction

Since 2012, the US Centers for Medicare & Medicaid Services (CMS) has aimed to incentivize networks of health care professionals and systems to contract together as Accountable Care Organizations (ACOs) to deliver coordinated high-quality care with lower health care spending for populations of Medicare fee-for-service beneficiaries.^1,2,3^ These alternative payment models, such as the Medicare Shared Savings Program (MSSP)—the largest Medicare ACO program—incentivize enhanced care coordination and delivery of high-value care, which may differentially affect care delivered to persons with dementia who have complex needs and often experience high-intensity and costly care at the end of life (EOL),^4,5,6,7,8,9,10^ care that may not be aligned with their preferences and goals.^11,12,13,14^

Prior research suggests that ACOs are associated with reductions in preventable emergency department (ED) visits,^15^ hospitalizations,^16^ and skilled nursing facility (SNF) length of stay^17^ for beneficiaries with dementia. Studies of the impact of ACOs on health care spending among a general population of Medicare beneficiaries estimate reductions in spending consistent with 1.4% to 4.9% savings but vary by entry year and ACO characteristics.^18,19,20^ In an early study of Medicare beneficiaries at the EOL, there were no differences observed in ED visits, hospitalizations, intensive care unit (ICU) admissions, or hospice days, and overall spending was similar between ACO and non-ACO.^21^ In a more recent study of Medicare beneficiaries with dementia who had a nursing home stay at the EOL, beneficiaries attributed to ACOs had higher adjusted odds of hospitalization in the last 30 days of life and hospice use but no evidence of differences in in-hospital death or invasive mechanical ventilation compared to traditional Medicare.^22^

To our knowledge, no studies have evaluated the impact of ACOs on EOL processes of care, outcomes, and health care spending among beneficiaries with dementia using a robust study design and recent data. To address this gap, we used difference-in-differences and event study designs to compare EOL care processes, outcomes, and health care spending between Medicare beneficiaries with dementia treated in MSSP ACO vs non-ACO.

## Methods

This study was reviewed and granted exempt by the University of California, Los Angeles Institutional Review Board. Informed consent was not required because this was secondary use of administrative data. This study was reported according to the Strengthening the Reporting of Observational Studies in Epidemiology (STROBE) reporting guideline.

### Data Source and Sample

We included Medicare fee-for-service beneficiaries who died from 2017 to 2020 and who were age 66 years or older with continuous Medicare Parts A and B coverage throughout the baseline year (ie, 1 year prior to year of death) and year of death (eFigure in Supplement 1). We used a 20% random sample of claims data based on the Master Beneficiary Summary File. Beneficiaries with dementia were identified in the baseline year using the Chronic Conditions Data Warehouse (CCW) algorithm for Alzheimer disease and related disorders or senile dementia, which has been previously validated.^23,24,25,26^ We excluded beneficiaries who (1) were unable to be attributed to a main taxpayer identification number or CMS certification number; (2) were attributed to an ACO in 2016, to only capture ACO entry from 2017 to 2019; (3) lived outside of the US; or (4) had missing data for a variable of interest (ie, residential zip code−level median annual household income, Hospital Referral Region [HRR]).

### ACO Status

Exposure was a binary indicator of whether a beneficiary was attributed to MSSP ACO (1-sided or 2-sided risk models) vs non-ACO. Similar to previous studies,^18,19,27^ we used evaluation and management claims from outpatient and carrier files in the baseline year (ie, to account for potential concerns about beneficiaries being attributed out of ACOs toward the EOL)^28^ to assign each beneficiary to an ACO vs non-ACO taxpayer identification number or CMS certification number that accounted for the most charges for primary care services by a primary care physician (ie, plurality). We required at least 1 outpatient visit or service with a primary care physician, similar to the MSSP Assignment Policy Step 1,^3^ by identifying only qualifying primary care services filed by a responsible physician with a primary care specialty (ie, family practice, general practice, geriatric medicine, or internal medicine). Using the MSSP ACO Provider-level Research Identifiable File for each ACO, we determined the ACO entry year (2017-2019). Even when attributed to an ACO, a beneficiary may still have received care outside of the ACO.

### EOL Care Processes and Outcomes

These EOL care processes and outcomes were evaluated: (1) billing for advance care planning (ACP) at any time in the data; (2) palliative care counseling in the last 6 months of life^29,30^; (3) hospice in the last 6 months of life; (4) high-intensity care in the last 30 days of life (ie, ED visit, hospitalization, ICU admission, in-hospital death, cardiopulmonary resuscitation [CPR] or mechanical ventilation, or feeding tube placement). See eTable 1 in Supplement 1 for outcome definitions.

### Health Care Spending

The outcomes included health care spending in the last 6 months of life. We calculated total health care spending as the sum of Medicare payments for services covered by Parts A or B, beneficiary payments for cost-sharing, and payment by a primary payer other than Medicare.^18,19,31^ We assessed health care spending by component claim categories: carrier, inpatient, outpatient, SNF, home health agency, and hospice.^21,32^ We did not include durable medical equipment or prescription drug spending.

### Adjustment Variables

We adjusted for beneficiary characteristics in the baseline year (ie, 1 year prior to year of death) except as noted below, including age at death (categorical); sex; race and ethnicity using the Research Triangle Institute race code^33,34^ (non-Hispanic Black [hereafter, Black], Hispanic, non-Hispanic White [hereafter, White], or Other [American Indian or Alaska Native, Asian or Pacific Islander, any other, or unknown]); long-term nursing home resident status, defined by a comprehensive or quarterly Minimum Data Set assessment in the last 90 days of life with entry date more than 100 days from assessment date^35,36,37^; residential zip code−level median annual household income (quintile); dual Medicare-Medicaid coverage; coexisting conditions (dummy variables for 25 nondementia-related CCW conditions; eTable 2 in Supplement 1); frailty based on at least 2 of 18 categories of claims-based surrogates used in a previously validated frailty index (binary) (eTable 3 in Supplement 1)^38,39,40,41^; and Hierarchical Condition Category (HCC) risk score.^18,19,27,42^

### Statistical Analysis

We used 2 approaches: (1) a difference-in-differences design to compare differential changes in EOL care processes and outcomes, and health care spending among beneficiaries attributed to ACO during the study period (2017-2020) vs the control group of beneficiaries who were never attributed to an ACO during the same period; and (2) an event study design to estimate differential changes over time for each outcome by relative year to ACO entry (2017-2019) compared to the control group of beneficiaries who were never attributed to an ACO. For difference-in-differences, each outcome was regressed on an indicator variable representing the interaction between being attributed to an ACO and the postintervention period to estimate the average treatment effect on the treated.^43,44,45^ For event study design, because we modeled beneficiaries receiving treatment at different times, we included indicator variables to represent combinations of ACO status and year of death relative to ACO entry year for 2017 to 2020.^45,46,47^ *Time zero* was defined as the year prior to ACO entry. The event study design formally tested the parallel trends assumption (ie, in the absence of ACO entry, outcomes for ACO and non-ACO groups would trend similarly over time) by evaluating whether the coefficients for the preintervention period (ie, relative years −2, −1, 0) were not statistically different from zero.

We used linear regression models for binary outcomes (ie, linear probability models) to estimate average effects rather than logistic regression, given the potential complete or quasi-separation issues with logistic regression, and for ease of interpretation of regression coefficients as percentage-point differential changes between ACO and non-ACO.^44,48^ All regression models were adjusted for beneficiary characteristics, included fixed effects for each unique ACO, HRR, year of death, and interaction of HRR and year of death, and were clustered at the ACO level for beneficiaries attributed to ACO and HRR level for non-ACO beneficiaries. We used the Holm-Bonferroni method to account for multiple comparisons and reported unadjusted and adjusted *P* values (*P* < .05 was considered statistically significant).^49,50^ We also examined the association between beneficiary attribution to an ACO and total health care spending and each component claim category by fitting multivariable ordinary least squares linear regression models with the same adjustments as above.

### Sensitivity Analyses

To test the robustness of our findings, we conducted additional analyses using inverse probability weighting (IPW), along with difference-in-differences design (ie, the doubly robust method given that we adjusted for confounders using IPW as well as in the regression models), with the aim of improving the balance in beneficiary characteristics between ACO and non-ACO. We fit a logistic model to predict attribution to ACO as a function of observed covariates of interest (ie, propensity score). Then, we calculated a propensity score weight for each beneficiary to represent the inverse of the probability of being attributed to the observed group. Statistical analyses were conducted using SAS Enterprise Guide, version 7.15 (SAS Institute), and Stata/MP, version 16.1 (StataCorp), from June 2023 to December 2024.

## Results

The 20% random sample analyzed included 162 034 Medicare beneficiaries (mean [SD] age, 85.0 [7.9] years; 94 304 female [58.2%] and 67 730 male [41.8%]; 12 581 Black [7.8%], 8 180 Hispanic [5.1%], 134 995 White [83.3%], and 6 278 individuals of other race and ethnicity [3.9%]) (Table 1). Of these, 51 191 (31.6%) were attributed to MSSP ACO; they were more likely to be younger, female, White, have a lower zip code-level median annual household income, have a lower HCC risk score, have cancer, have a later year of death date; and less likely to have diabetes, heart failure, dual Medicare-Medicaid coverage, or be a long-term nursing home resident compared to non-ACO beneficiaries (Table 1).

**Table 1.** Baseline Characteristics of Medicare Beneficiaries With Dementia Who Died From 2017 to 2020, by Accountable Care Organization (ACO) Status^a^

Characteristic	No. (%)		
	Total	ACO status	
		ACO	Non-ACO
Beneficiaries, No.	162 034	51 191	110 843
Age, mean (SD), y	85.0 (7.9)	84.9 (7.8)	85.0 (7.9)
Sex			
Female	94 304 (58.2)	29 985 (58.6)	64 319 (58.0)
Male	67 730 (41.8)	21 206 (41.4)	46 524 (42.0)
Race and ethnicity^b^			
Black	12 581 (7.8)	3769 (7.4)	8812 (8.0)
Hispanic	8180 (5.1)	2327 (4.6)	5853 (5.3)
White	134 995 (83.3)	43 748 (85.5)	91 247 (82.3)
Other^b^	6278 (3.9)	1347 (2.6)	4931 (4.5)
Annual household income, mean (SD), $^c^	66 457 (27 659)	66 069 (26 964)	66 636 (27 973)
Dual Medicare-Medicaid coverage	21 514 (13.3)	6179 (12.1)	15 335 (13.8)
Long-term nursing home resident	14 210 (8.8)	4332 (8.5)	9878 (8.9)
Selected coexisting conditions^d^			
Chronic kidney disease	89 882 (55.5)	28 490 (55.7)	61 392 (55.4)
Heart failure	74 327 (45.9)	23 139 (45.2)	51 188 (46.2)
Diabetes	64 830 (40.0)	19 893 (38.9)	44 937 (40.5)
COPD	46 553 (28.7)	14 747 (28.8)	31 806 (28.7)
Cancer	24 950 (15.4)	8034 (15.7)	16 916 (15.3)
Coexisting conditions, mean (SD), No.^d^	6.5 (3.1)	6.5 (3.1)	6.5 (3.1)
Frailty^e^	117 814 (72.7)	37 283 (72.8)	80 531 (72.7)
HCC risk score, mean (SD)	3.2 (2.1)	3.2 (2.1)	3.2 (2.2)
Year of death			
2017	39 781 (24.6)	11 996 (23.4)	27 785 (25.1)
2018	39 693 (24.5)	12 442 (24.3)	27 251 (24.6)
2019	38 691 (23.9)	12 406 (24.2)	26 285 (23.7)
2020	43 869 (27.1)	14 347 (28.0)	29 522 (26.6)

Abbreviations: COPD, chronic obstructive pulmonary disease; HCC, hierarchical condition category.

^a^ Study population comprised a 20% random sample of eligible beneficiaries. Characteristics were measured in the baseline year prior to the year of death except for long-term nursing home resident status, which was determined by the most recent Minimum Data Set assessment within 90 days before death.

^b^ Data were collected from Research Triangle Institute race code variable in the Medicare Master Beneficiary Summary File; Other included American Indian or Alaska Native, Asian or Pacific Islander, any other, and unknown.

^c^ Per median zip code−level annual household income.

^d^ Indicators for coexisting conditions included 25 nondementia-related Chronic Conditions Data Warehouse conditions and count excluded cataracts and glaucoma.

^e^ Based on having at least 2 categories of claims-based surrogates of frailty.

### End-of-Life Care Processes and Outcomes

Table 2 shows the differential changes in EOL care processes and outcomes for ACO beneficiaries compared with the control beneficiaries who were never attributed to ACO (difference-in-differences estimates). In difference-in-differences analyses, we found no evidence of change in proportions of beneficiaries who had billing for ACP, palliative care counseling in the last 6 months of life, hospice in the last 6 months of life, or high-intensity care in the last 30 days of life (ie, ED visit, hospitalization, ICU admission, in-hospital death, CPR or mechanical ventilation, or feeding tube placement) between ACO and non-ACO.

**Table 2.** Differential Changes in End-of-Life Care Processes, Outcomes, and Spending for Medicare Accountable Care Organization (ACO) Beneficiaries Compared With Control Group^a^

End-of-life care	ACO beneficiaries, baseline mean, %^b^	Difference-in-differences estimate, pp (95% CI)	*P* value	Adjusted *P* value^c^
Advance care planning	15.2	0.3 (−0.7 to 1.2)	.56	>.99
Palliative care counseling^d^	17.8	−0.5 (−1.3 to 0.3)	.20	>.99
Hospice^d^	65.5	−0.4 (−1.4 to 0.5)	.38	>.99
ED visit^e^	53.1	−0.6 (−1.7 to 0.5)	.29	>.99
Hospitalization^e^	45.2	−0.7 (−1.9 to 0.4)	.22	>.99
ICU admission^e^	22.7	−0.2 (−1.1 to 0.7)	.63	>.99
In-hospital death	14.9	−0.5 (−1.2 to 0.3)	.22	>.99
CPR or mechanical ventilation^e^	10.4	0 (−0.6 to 0.6)	.98	.98
Feeding tube placement^e^	1.3	0.1 (−0.1 to 0.4)	.24	>.99
Total health care spending, $^d^	41 716	−632 (−1377 to 113)	.10	.96

Abbreviations: CPR, cardiopulmonary resuscitation; ED, emergency department; ICU, intensive care unit; pp, percentage points.

^a^ Data of a 20% random sample of Medicare beneficiaries (age ≥66 years) with dementia who died from 2017 to 2020, attributed to ACO vs non-ACO. Linear probability (binary outcomes) and linear regression (spending) models were adjusted for age, sex, race and ethnicity, long-term nursing home resident status, median zip code−level annual household income, dual Medicare-Medicaid coverage, coexisting conditions, frailty, and hierarchical condition category risk score, and included fixed effects for each unique ACO, hospital referral region (HRR), year of death, and interaction of HRR and year of death, and clustered at the ACO level for ACO beneficiaries and HRR level for non-ACO beneficiaries.

^b^ Calculated as the unadjusted mean prior to ACO entry among beneficiaries attributed to ACO.

^c^ *P* values were adjusted with the Holm-Bonferroni method to account for multiple comparisons (adjusted *P* < .05 is statistically significant).

^d^ During last 6 months of life.

^e^ During last 30 days of life.

The Figure and eTable 4 in Supplement 1 show the differential changes using the event study design, and similarly, we found no evidence that EOL care processes or outcomes changed after ACO entry. For the formal test of the parallel trends assumption, although the coefficient for differential change in proportion of ICU admissions for ACO relative year −1 was greater than zero and for proportion of CPR or mechanical ventilation for ACO relative year −2 was less than zero, these were not statistically significant when adjusted for multiple comparisons. Moreover, we found no evidence of systematic monotonic patterns over time, eg, linear and monotonically increasing trends in regression coefficients during the preintervention period. Thus, this should not have limited the analysis nor the interpretation of the results as null findings.

**Figure.**
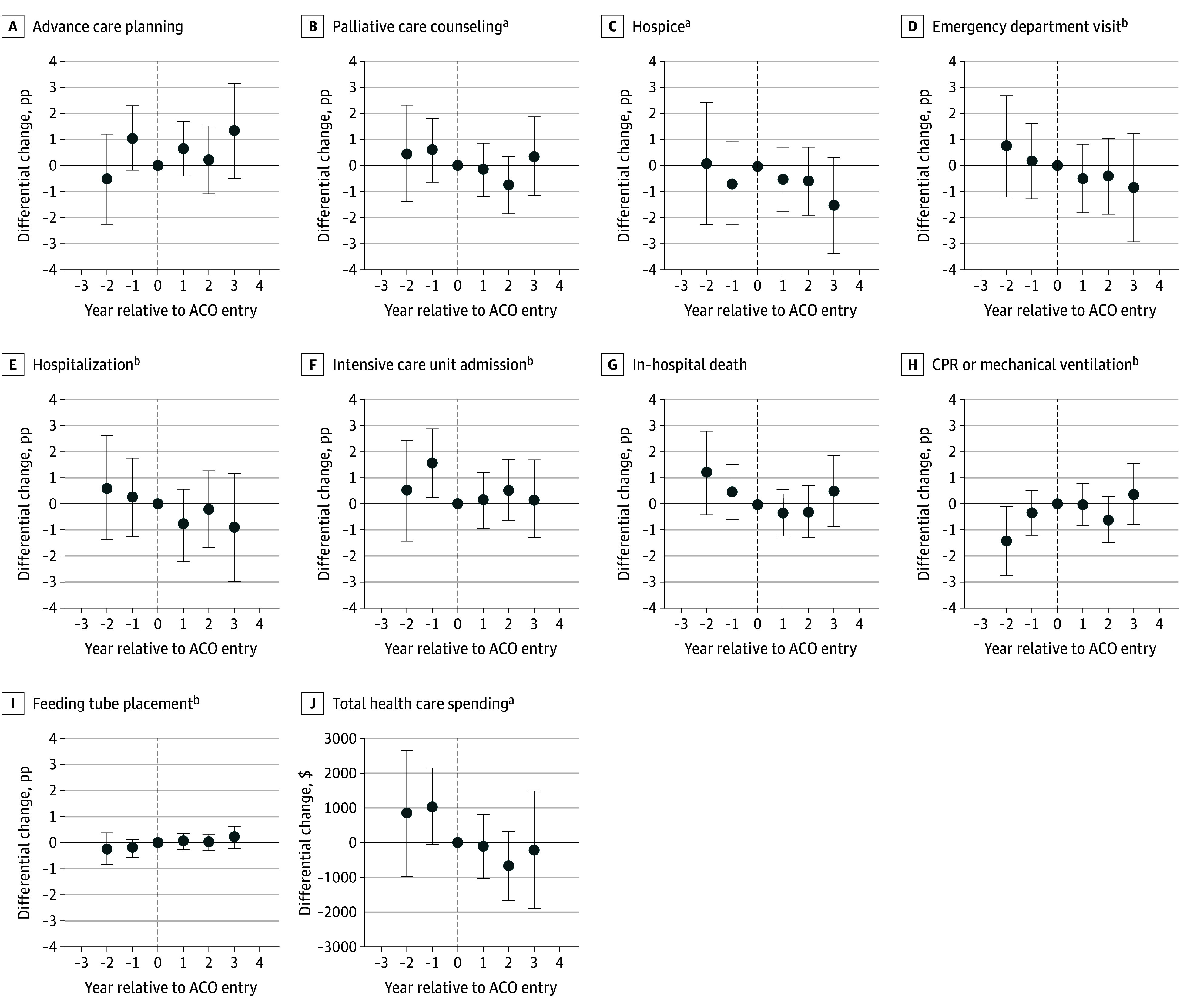
Differential Changes in End-of-Life Care Processes, Outcomes, and Spending for Medicare Accountable Care Organization (ACO) Beneficiaries Compared With Control Group, Using the Event Study Design *Time zero* was defined as the year before ACO entry, represented by the dashed line; error bars indicate 95% CIs. CPR indicates cardiopulmonary resuscitation, and pp, percentage points. ^a^During the last 6 months of life. ^b^During the last 30 days of life.

### Health Care Spending

Table 2 and eTable 5 in Supplement 1 show the differential changes in health care spending in the last 6 months of life for total and component claim categories, respectively (difference-in-differences estimates). Results from the event study design for total EOL health care spending were similar (Figure). We found no evidence that EOL health care spending differed between ACO vs non-ACO beneficiaries.

### Sensitivity Analyses

The sensitivity analyses using IPW to balance baseline beneficiary characteristics (eTable 6 in Supplement 1) showed similar results for difference-in-differences analyses (eTable 7 in Supplement 1) compared to the main analyses.

## Discussion

Using nationally representative data of Medicare fee-for-service beneficiaries with dementia, we found no evidence that EOL care processes, outcomes, or spending differed between beneficiaries treated in ACOs vs non-ACOs. Overall, the data suggest that for persons with dementia at the EOL, ACOs may not be differentially affecting costly inpatient and SNF care nor outpatient and home health services compared to non-ACO care.

There are multiple possible explanations for these null findings. In this broad population of beneficiaries with dementia at the EOL, we found no evidence of difference in billing rates for ACP, palliative care counseling, or hospice with ACOs, which could represent potential opportunities for processes toward less inpatient and costly EOL care.^27,51,52^ Although ACOs aim to reduce preventable ED visits,^53^ we found no evidence of difference in proportion of ED visits among ACO beneficiaries compared with non-ACO. This may be due to proactive outreach by beneficiaries and health systems in ACOs, which could lead to ED presentations regardless of whether stabilization and discharge from the ED represents lower quality of care, but may also reflect insufficient ED alternatives^15^ or fragmentation of care.^28^ Although ACO quality indicators include preventive care and care coordination,^53,54^ current incentives may not be strong enough for individualized care for persons with dementia,^28^ EOL care,^27,52^ or assessment of how care aligns with goals and preferences.^55,56^

As the sensitivity analyses using IPW suggest, our null findings for EOL care processes, outcomes, and health care spending are independent of beneficiary characteristics that could be associated with attribution to ACOs. Although not explored in this study, there may be heterogeneity in processes and outcomes of care or how ACOs are able to deliver and coordinate care related to ACO characteristics, such as number of beneficiaries that mask potential differences when analyzed at the individual ACO level. Changes to patient care also depend on clinician practice.^57,58,59^ More recent alternative payment models with quality indicators individualized for beneficiaries with dementia and their caregivers, such as the CMS Guiding an Improved Dementia Experience (GUIDE) Model, may better incentivize health system support for outpatient care.^60^

This study found no evidence of difference in health care spending at the EOL between ACO compared to non-ACO beneficiaries. These study findings are consistent with those of prior studies on EOL care processes, which found small and likely unsubstantial changes associated with ACO entry that varied between 2012 and 2014.^27^ In addition, prior studies have found small changes in annual spending by ACO status for Medicare beneficiaries,^18,19^ and no evidence of differences in EOL spending for beneficiaries who died in 2012 or 2015^21^ and who died with cancer.^32^ While a savings of up to 4.9% was observed in a previous study of Medicare beneficiaries for physician-group ACOs that entered in 2012,^19^ this was higher than for other entry years and when compared to hospital-integrated ACOs. Although we were unable to observe mechanisms for spending in ACOs vs non-ACO, it is possible—especially at the EOL that current processes do not align beneficiaries’ goals and preferences for less high-intensity care with differences in health care utilization and thus spending, and that ACOs are currently unable to affect beneficiary choice or refer beneficiaries to sources of care that may be more aligned with the ACO network’s mission to deliver coordinated and efficient care.^52^

Although previous studies suggest that ACOs are associated with lower odds of preventable ED visits,^15^ reductions in hospitalizations,^16^ and shorter SNF length of stay^17^ for persons with dementia, these studies have been mostly observational and used cross-sectional data. Results from the few studies of other clinical conditions on the association of ACOs with EOL care and health care spending have varied. In the first few years of ACO implementation, among Medicare beneficiaries at the EOL, there was no evidence of differences in ED visits, hospitalizations, ICU admissions, hospice days, or overall spending.^21^ A study of Medicare beneficiaries with cancer at the EOL found no meaningful differences in health care utilization or spending, including in ED visits, hospitalization, ICU admission, and hospice use.^32^ In a recent study of Medicare beneficiaries with dementia and a nursing home stay at the EOL, beneficiaries attributed to ACOs had higher adjusted odds of hospitalization and hospice use but not in-hospital death or invasive mechanical ventilation compared to traditional Medicare.^22^ Although prior studies suggest that ACOs may be associated with small reductions in spending without observably worse quality, our overall findings are consistent in suggesting that ACOs may have little impact on EOL care processes, outcomes, and health care spending.^20^

### Limitations

The study’s findings should be interpreted in the context of its limitations. First, dementia was determined by the CCW algorithm and may have been subject to misclassification bias; however, previous studies have validated the use of Medicare claims data to identify persons with dementia, and prevalence of dementia diagnosis in our sample was similar across the years of our study period.^24,25,26^ We did not assess dementia severity; however, we adjusted for CCW conditions, frailty, and HCC risk score as proxies for overall illness severity. Second, we were unable to observe unbilled ACP discussions or palliative care counseling.^61^ Third, high-intensity care at the EOL was defined as the last 30 days of life, consistent with prior research.^22,62^ Although dementia is a chronic condition with potentially difficult prognosis of timing of the EOL, we do not expect bias in our findings between ACO and non-ACO because our sample only included deceased beneficiaries. Fourth, there may be remaining unobserved confounding. For example, beneficiaries in ACO vs non-ACO may have more exposure to physician factors, such as geriatrics-trained physicians, more goal-concordant care, and thus, bias toward an overestimate of the association between ACOs and EOL care processes and outcomes^63^; however, we found no evidence that outcomes differed by ACO status. Lastly, these findings may not be generalizable to younger or Medicare Advantage populations.^22,64^

## Conclusions

Using nationally representative data on Medicare beneficiaries with dementia at the EOL, this quasi-experimental study found no evidence that EOL care processes, outcomes, or health care spending changed after ACO entry for beneficiaries attributed to ACOs compared to non-ACO. Overall, these data suggest that alternative payment models to ACOs may be needed to coordinate high-quality care with lower health care spending for beneficiaries with dementia at the EOL.

## Notes

Supplement 1. eFigure. Flowchart of beneficiaries eTable 1. Outcome definitions eTable 2. Chronic Conditions Data Warehouse conditions eTable 3. Frailty and functional impairment index eTable 4. Differential changes in end-of-life care processes, outcomes, and spending for ACO beneficiaries, as compared with the control group (using the event study design) eTable 5. Differential changes in end-of-life health care spending for ACO beneficiaries, as compared with the control group (difference-in-differences estimates) eTable 6. Characteristics of the study population at baseline using inverse probability weighting eTable 7. Differential changes in end-of-life care processes, outcomes, and spending for ACO beneficiaries, as compared with the control group (difference-in-differences estimates with inverse probability weighting)

Supplement 2. Data Sharing Statement

## References

[1] US Centers for Medicare & Medicaid Services. Accountable Care Organizations (ACOs). General Information. Accessed May 25, 2024. https://www.cms.gov/priorities/innovation/innovation-models/aco

[2] US Centers for Medicare & Medicaid Services. Final Rule Creates Pathways to Success for the Medicare Shared Savings Program. Accessed May 27, 2024. https://www.cms.gov/newsroom/fact-sheets/final-rule-creates-pathways-success-medicare-shared-savings-program

[3] US Centers for Medicare & Medicaid Services. Medicare Shared Savings Program Shared Savings and Losses and Assignment Methodology Specifications. Published online 2017. Accessed May 27, 2024. https://www.cms.gov/Medicare/Medicare-Fee-for-Service-Payment/sharedsavingsprogram/Downloads/Shared-Savings-Losses-Assignment-Spec-V5.pdf

[4] Mitchell SL, Morris JN, Park PS, Fries BE. Terminal care for persons with advanced dementia in the nursing home and home care settings. J Palliat Med. 2004;7(6):808-816. doi:10.1089/jpm.2004.7.808 15684848

[5] Mitchell SL, Kiely DK, Hamel MB. Dying with advanced dementia in the nursing home. Arch Intern Med. 2004;164(3):321-326. doi:10.1001/archinte.164.3.321 14769629

[6] Mitchell SL, Teno JM, Kiely DK, . The clinical course of advanced dementia. N Engl J Med. 2009;361(16):1529-1538. doi:10.1056/NEJMoa0902234 19828530 PMC2778850

[7] Cai S, Gozalo PL, Mitchell SL, . Do patients with advanced cognitive impairment admitted to hospitals with higher rates of feeding tube insertion have improved survival? J Pain Symptom Manage. 2013;45(3):524-533. doi:10.1016/j.jpainsymman.2012.02.007 22871537 PMC3594461

[8] Teno JM, Gozalo P, Trivedi AN, . Site of death, place of care, and health care transitions among US Medicare beneficiaries, 2000-2015. JAMA. 2018;320(3):264-271. doi:10.1001/jama.2018.8981 29946682 PMC6076888

[9] Crouch E, Probst JC, Bennett K, Eberth JM. Differences in Medicare utilization and expenditures in the last six months of life among patients with and without Alzheimer’s disease and related disorders. J Palliat Med. 2019;22(2):126-131. doi:10.1089/jpm.2018.0147 30328762

[10] Keeney T, Belanger E, Jones RN, Joyce NR, Meyers DJ, Mor V. High-Need Phenotypes in Medicare Beneficiaries: Drivers of Variation in Utilization and Outcomes. J Am Geriatr Soc. 2020;68(1):70-77. doi:10.1111/jgs.16146 31454082 PMC6952536

[11] Volandes AE, Paasche-Orlow MK, Barry MJ, . Video decision support tool for advance care planning in dementia: randomised controlled trial. BMJ. 2009;338:b2159. doi:10.1136/bmj.b2159 19477893 PMC2688013

[12] Mitchell SL, Palmer JA, Volandes AE, Hanson LC, Habtemariam D, Shaffer ML. Level of care preferences among nursing home residents with advanced dementia. J Pain Symptom Manage. 2017;54(3):340-345. doi:10.1016/j.jpainsymman.2017.04.020 28797857 PMC5744260

[13] Tjia J, D’Arcangelo N, Carlston D, . US clinicians’ perspectives on advance care planning for persons with dementia: a qualitative study. J Am Geriatr Soc. 2023;71(5):1473-1484. doi:10.1111/jgs.18197 36547969 PMC10175113

[14] Zhu Y, Olchanski N, Cohen JT, . Life-sustaining treatments among Medicare beneficiaries with and without dementia at the end of life. J Alzheimers Dis. 2023;96(3):1183-1193. doi:10.3233/JAD-230692 37955089 PMC10777481

[15] Wang N, Amaize A, Chen J. Accountable Care hospitals and preventable emergency department visits for rural dementia patients. J Am Geriatr Soc. 2021;69(1):185-190. doi:10.1111/jgs.16858 33026671 PMC8276835

[16] Chen J, Benjenk I, Barath D, Anderson AC, Reynolds CF III. Disparities in preventable hospitalization among patients with Alzheimer diseases. Am J Prev Med. 2021;60(5):595-604. doi:10.1016/j.amepre.2020.12.014 33832801 PMC8068589

[17] Bynum JPW, Montoya A, Lawton EJ, . Accountable Care Organization attribution and post-acute skilled nursing facility outcomes for people living with dementia. J Am Med Dir Assoc. 2024;25(1):53-57.e2. doi:10.1016/j.jamda.2023.10.031 38081322 PMC11613903

[18] McWilliams JM, Hatfield LA, Chernew ME, Landon BE, Schwartz AL. Early performance of Accountable Care Organizations in Medicare. N Engl J Med. 2016;374(24):2357-2366. doi:10.1056/NEJMsa1600142 27075832 PMC4963149

[19] McWilliams JM, Hatfield LA, Landon BE, Hamed P, Chernew ME. Medicare spending after 3 years of the Medicare Shared Savings Program. N Engl J Med. 2018;379(12):1139-1149. doi:10.1056/NEJMsa1803388 30183495 PMC6269647

[20] Wilson M, Guta A, Waddell K, Lavis J, Reid R, Evans C. The impacts of accountable care organizations on patient experience, health outcomes and costs: a rapid review. J Health Serv Res Policy. 2020;25(2):130-138. doi:10.1177/1355819620913141 32321282

[21] Lam MB, Friend TH, Erfani P, Orav EJ, Jha AK, Figueroa JF. ACO spending and utilization among Medicare patients at the end of life: an observational study. J Gen Intern Med. 2022;37(13):3275-3282. doi:10.1007/s11606-021-07183-9 35022958 PMC9550919

[22] Teno JM, Keohane LM, Mitchell SL, . Dying with dementia in Medicare Advantage, Accountable Care Organizations, or traditional Medicare. J Am Geriatr Soc. 2021;69(10):2802-2810. doi:10.1111/jgs.17225 33989430 PMC8497397

[23] Chronic Conditions Data Warehouse. 27 CCW Chronic Conditions Algorithms: MBSF_CC{YYYY} File. February 2023. Accessed July 23, 2024. https://www2.ccwdata.org/documents/10280/19139421/ccw-chronic-condition-algorithms.pdf

[24] Taylor DH Jr, Fillenbaum GG, Ezell ME. The accuracy of medicare claims data in identifying Alzheimer’s disease. J Clin Epidemiol. 2002;55(9):929-937. doi:10.1016/S0895-4356(02)00452-3 12393082

[25] Taylor DH Jr, Østbye T, Langa KM, Weir D, Plassman BL. The accuracy of Medicare claims as an epidemiological tool: the case of dementia revisited. J Alzheimers Dis. 2009;17(4):807-815. doi:10.3233/JAD-2009-1099 19542620 PMC3697480

[26] Chen Y, Tysinger B, Crimmins E, Zissimopoulos JM. Analysis of dementia in the US population using Medicare claims: insights from linked survey and administrative claims data. Alzheimers Dement (N Y). 2019;5:197-207. doi:10.1016/j.trci.2019.04.003 31198838 PMC6556828

[27] Gilstrap LG, Huskamp HA, Stevenson DG, Chernew ME, Grabowski DC, McWilliams JM. Changes in end-of-life care in the Medicare Shared Savings Program. Health Aff (Millwood). 2018;37(10):1693-1700. doi:10.1377/hlthaff.2018.0491 30273040 PMC6233308

[28] Johnston KJ, Loux T, Joynt Maddox KE. Risk selection and care fragmentation at Medicare Accountable Care Organizations for patients with dementia. Med Care. 2023;61(8):570-578. doi:10.1097/MLR.0000000000001876 37411003 PMC10328553

[29] Walling A, Lorenz KA, Dy SM, . Evidence-based recommendations for information and care planning in cancer care. J Clin Oncol. 2008;26(23):3896-3902. doi:10.1200/JCO.2007.15.9509 18688058

[30] Walling AM, Ahluwalia SC, Wenger NS, ; Palliative Care Cirrhosis Quality Expert Panel. Palliative care quality indicators for patients with end-stage liver disease due to cirrhosis. Dig Dis Sci. 2017;62(1):84-92. doi:10.1007/s10620-016-4339-3 27804005 PMC5384571

[31] Chronic Conditions Data Warehouse. Technical Guidance. Getting Started with CMS Medicare Administrative Research Files. Published online September 2022. https://www2.ccwdata.org/web/guest/home/

[32] Lam MB, Zheng J, Orav EJ, Jha AK. Early Accountable Care Organization results in end-of-life spending among cancer patients. J Natl Cancer Inst. 2019;111(12):1307-1313. doi:10.1093/jnci/djz033 30859226 PMC6910163

[33] Bonito A, Eicheldinger C, Evensen C. Health Disparities: Measuring Health Care Use and Access for Racial/Ethnic Populations. RTI International; 2005.

[34] Eicheldinger C, Bonito A. More accurate racial and ethnic codes for Medicare administrative data. Health Care Financ Rev. 2008;29(3):27-42. 18567241 PMC4195038

[35] Hunnicutt JN, Tjia J, Lapane KL. Hospice use and pain management in elderly nursing home residents with cancer. J Pain Symptom Manage. 2017;53(3):561-570. doi:10.1016/j.jpainsymman.2016.10.369 28042063 PMC5337160

[36] Goodwin JS, Li S, Zhou J, Graham JE, Karmarkar A, Ottenbacher K. Comparison of methods to identify long term care nursing home residence with administrative data. BMC Health Serv Res. 2017;17(1):376. doi:10.1186/s12913-017-2318-9 28558756 PMC5450097

[37] US Centers for Medicare & Medicaid Services. MDS 3.0 Quality Measures User’s Manual (V12.1); 2019. https://www.cms.gov/Medicare/Quality-Initiatives-Patient-Assessment-Instruments/NursingHomeQualityInits/Downloads/MDS-30-QM-USERS-MANUAL-v121.pdf

[38] Joynt KE, Figueroa JF, Beaulieu N, Wild RC, Orav EJ, Jha AK. Segmenting high-cost Medicare patients into potentially actionable cohorts. Healthc (Amst). 2017;5(1-2):62-67. doi:10.1016/j.hjdsi.2016.11.002 27914968

[39] Figueroa JF, Joynt Maddox KE, Beaulieu N, Wild RC, Jha AK. Concentration of potentially preventable spending among high-cost Medicare subpopulations: an observational study. Ann Intern Med. 2017;167(10):706-713. doi:10.7326/M17-0767 29049488

[40] Kim DH, Schneeweiss S. Measuring frailty using claims data for pharmacoepidemiologic studies of mortality in older adults: evidence and recommendations. Pharmacoepidemiol Drug Saf. 2014;23(9):891-901. doi:10.1002/pds.3674 24962929 PMC4149846

[41] Festa N, Shi SM, Kim DH. Accuracy of diagnosis and health service codes in identifying frailty in Medicare data. BMC Geriatr. 2020;20(1):329. doi:10.1186/s12877-020-01739-w 32894057 PMC7487915

[42] Pope GC, Kautter J, Ellis RP, . Risk adjustment of Medicare capitation payments using the CMS-HCC model. Health Care Financ Rev. 2004;25(4):119-141. 15493448 PMC4194896

[43] Roth J, Sant’Anna PHC, Bilinski A, Poe J. What’s trending in difference-in-differences? A synthesis of the recent econometrics literature. J Econom. 2023;235(2):2218-2244. doi:10.1016/j.jeconom.2023.03.008

[44] Miller S, Wherry LR. Health and access to care during the first 2 years of the ACA Medicaid expansions. N Engl J Med. 2017;376(10):947-956. doi:10.1056/NEJMsa1612890 28273021

[45] Miller S, Johnson N, Wherry LR. Medicaid and mortality: new evidence from linked survey and administrative data. Q J Econ. 2021;136(3):1783-1829. doi:10.1093/qje/qjab004

[46] Borusyak K, Jaravel X, Spiess J. Revisiting Event-Study Designs: Robust and Efficient Estimation. The Review of Economic Studies. Oxford Academic. Accessed December 16, 2024. https://academic.oup.com/restud/article/91/6/3253/7601390

[47] Miller DL. An introductory guide to event study models. J Econ Perspect. 2023;37(2):203-230. doi:10.1257/jep.37.2.203

[48] Hellevik O. Linear versus logistic regression when the dependent variable is a dichotomy. Qual Quant. 2009;43:59-74. doi:10.1007/s11135-007-9077-3

[49] Giacalone M, Agata Z, Cozzucoli PC, Alibrandi A. Bonferroni-Holm and permutation tests to compare health data: methodological and applicative issues. BMC Med Res Methodol. 2018;18(1):81. doi:10.1186/s12874-018-0540-8 30029629 PMC6054729

[50] Holm S. A Simple Sequentially Rejective Multiple Test Procedure. *Scand J Stat*. 1979. Accessed May 27, 2024. https://www.semanticscholar.org/paper/A-Simple-Sequentially-Rejective-Multiple-Test-Holm/b0ebbcf713b3ddf3f94325bc58dc39ff76fdc412

[51] Nicholas LH, Bynum JPW, Iwashyna TJ, Weir DR, Langa KM. Advance directives and nursing home stays associated with less aggressive end-of-life care for patients with severe dementia. Health Aff (Millwood). 2014;33(4):667-674. doi:10.1377/hlthaff.2013.1258 24711329 PMC4159465

[52] Driessen J, West T. Variation in End-Of-Life Care Is an Open Invitation for Accountable Care Organization Innovation. Health Affairs. https://www.healthaffairs.org/content/forefront/variation-end-of-life-care-open-invitation-accountable-care-organization-innovation

[53] Lewis VA, Schoenherr K, Fraze T, Cunningham A. Clinical coordination in accountable care organizations: A qualitative study. Health Care Manage Rev. 2019;44(2):127-136. doi:10.1097/HMR.0000000000000141 27926614 PMC5461217

[54] US Centers for Medicare & Medicaid Services. Medicare Shared Savings Program Quality Measure Benchmarks for the 2016 and 2017 Reporting Years. https://www.cms.gov/files/document/medicare-shared-savings-program-quality-measure-benchmarks-2016-and-2017-reporting-years-pdf.pdf

[55] Bleser WK, Saunders RS, Winfield L, . ACO serious illness care: survey and case studies depict current challenges and future opportunities. Health Aff (Millwood). 2019;38(6):1011-1020. doi:10.1377/hlthaff.2019.00013 31158012

[56] Bunker JN, Mitchell SL, Belanger E, Gozalo PL, Teno JM. Pain impacting quality of life in persons with dementia dying in the nursing home by Alternative Medicare Payment Model. J Palliat Med. 2022;25(12):1795-1801. doi:10.1089/jpm.2022.0047 35675641 PMC9784608

[57] Lally KM, Ducharme CM, Roach RL, Towey C, Filinson R, Tuya Fulton A. Interprofessional training: geriatrics and palliative care principles for primary care teams in an ACO. Gerontol Geriatr Educ. 2019;40(1):121-131. doi:10.1080/02701960.2018.1459595 29630470

[58] Markovitz AA, Rozier MD, Ryan AM, . Low-value care and clinician engagement in a large Medicare shared savings program ACO: a survey of frontline clinicians. J Gen Intern Med. 2020;35(1):133-141. doi:10.1007/s11606-019-05511-8 31705479 PMC6957659

[59] Teno JM, Mitchell S, Belanger E, Bunker J, Gozalo PL. Accountable Care Organizations (ACOs) could potentially improve the quality of care in those afflicted with dementia. J Pain Symptom Manage. 2021;62(2):e1-e2. doi:10.1016/j.jpainsymman.2021.04.003 33957253 PMC8500342

[60] US Centers for Medicare & Medicaid Services. Guiding an Improved Dementia Experience (GUIDE) Model. Accessed May 30, 2024. https://www.cms.gov/priorities/innovation/innovation-models/guide

[61] Gotanda H, Walling AM, Zhang JJ, Xu H, Tsugawa Y. Timing and setting of billed advance care planning among Medicare decedents in 2017-2019. J Am Geriatr Soc. 2023;71(10):3237-3243. doi:10.1111/jgs.18476 37335260 PMC10592584

[62] Earle CC, Neville BA, Landrum MB, . Evaluating claims-based indicators of the intensity of end-of-life cancer care. Int J Qual Health Care. 2005;17(6):505-509. doi:10.1093/intqhc/mzi061 15985505

[63] Gotanda H, Zhang JJ, Reuben DB, . Association between physicians’ geriatric training and patterns of end-of-life care delivered to persons with dementia. J Am Geriatr Soc. 2023;71(11):3457-3466. doi:10.1111/jgs.18510 37470082 PMC10799178

[64] Nicholas LH, Fischer SM, Arbaje AI, Perraillon MC, Jones CD, Polsky D. Medicare-covered services near the end of life in Medicare Advantage vs traditional Medicare. JAMA Health Forum. 2024;5(7):e241777. doi:10.1001/jamahealthforum.2024.1777 39028655 PMC11259900

