# Supplementary material for: End-of-Life Care and Health Care Spending for Medicare Beneficiaries With Dementia in Accountable Care Organizations
Source: JAMA Health Forum. 2025 May 9;6(5):e250731. doi: 10.1001/jamahealthforum.2025.0731 (PMC12065038; doi:10.1001/jamahealthforum.2025.0731)
Supplement: Supplement 1. — eFigure. Flowchart of beneficiaries eTable 1. Outcome definitions eTable 2. Chronic Conditions Data Warehouse conditions eTable 3. Frailty and functional impairment index eTable 4. Differential changes in end-of-life care processes, outcomes, and spending for ACO beneficiaries, as compared with the control group (using the event study design) eTable 5. Differential changes in end-of-life health care spending for ACO beneficiaries, as compared with the control group (difference-in-differences estimates) eTable 6. Characteristics of the study population at baseline using inverse probability weighting eTable 7. Differential changes in end-of-life care processes, outcomes, and spending for ACO beneficiaries, as compared with the control group (difference-in-differences estimates with inverse probability weighting) [file jamahealthforum-e250731-s001.pdf]

Supplemental Online Content

Zhang JJ, Reuben DB, Walling AM, et al. End-of-Life Care and Health Care Spending for Medicare Beneficiaries With Dementia in Accountable Care Organizations. *JAMA Health Forum.* Published online May 9, 2025. doi:10.1001/jamahealthforum.2025.0731

eFigure. Flowchart of beneficiaries

eTable 1. Outcome definitions

eTable 2. Chronic Conditions Data Warehouse conditions

eTable 3. Frailty and functional impairment index

eTable 4. Differential changes in end-of-life care processes, outcomes, and spending for ACO beneficiaries, as compared with the control group (using the event study design)

eTable 5. Differential changes in end-of-life health care spending for ACO beneficiaries, as compared with the control group (difference-in-differences estimates)

eTable 6. Characteristics of the study population at baseline using inverse probability weighting

eTable 7. Differential changes in end-of-life care processes, outcomes, and spending for ACO beneficiaries, as compared with the control group (difference-in-differences estimates with inverse probability weighting)

This supplemental material has been provided by the authors to give readers additional information about their work.

© 2025 Zhang JJ et al. *JAMA Health Forum*. All rights reserved, including those for text and data mining, AI training, and similar technologies.

eFigure. Flowchart of beneficiaries

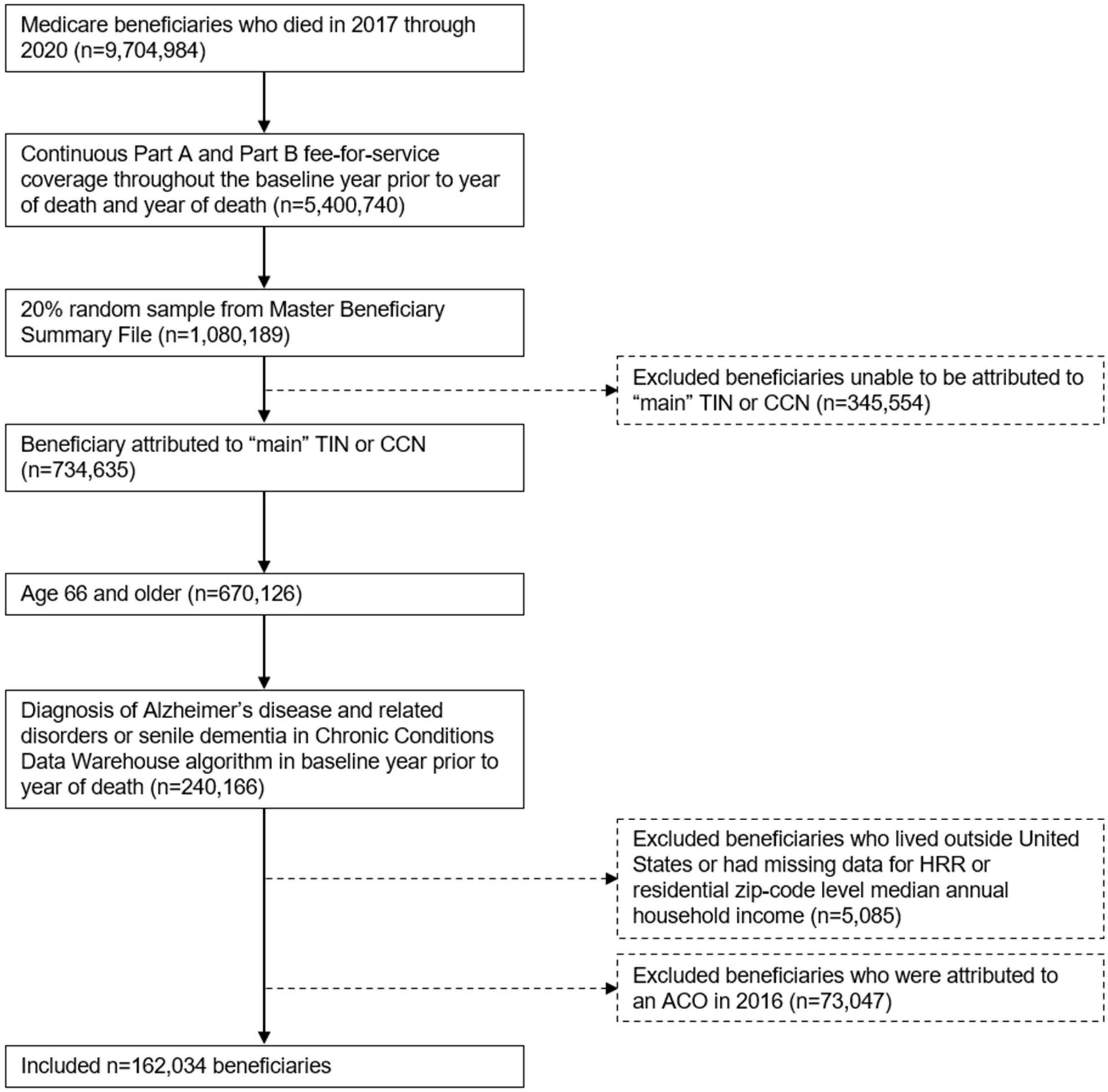

Abbreviations: ACO = Accountable care Organization, CCN = Centers for Medicare and Medicaid Services (CMS) Certification Number, HRR = Hospital Referral Region, TIN = Taxpayer Identification Number.

© 2025 Zhang JJ et al. *JAMA Health Forum*. All rights reserved, including those for text and data mining, AI training, and similar technologies.

eTable 1. Outcome definitions

Outcome	Definition
Billed advance care planning	CPT codes: 99497, 99498 in Carrier file
Palliative care counseling	Palliative care counseling ICD-10 code: Z51.5 in Carrier file
Hospice	Hospice use: presence of Claim From Date (CLM_FROM_DT) in Hospice file
Emergency department visit	Revenue Center Code (REV_CNTR) = 0450–0459, 0981 in Inpatient file or Outpatient file
Hospitalization	Beneficiary Discharge Date (NCH_BENE_DSCHRG_DT) in Inpatient file
Intensive care unit admission	REV_CNTR = 0200-0209 in Inpatient file
In-hospital death	Patient Discharge Status Code (PTNT_DSCHRG_STUS_CD) = Expired (patient did not recover)
Cardiopulmonary resuscitation or mechanical ventilation	Cardiopulmonary resuscitation (and/or defibrillation) ICD-10 codes: 5A12012, 5A19054, 5A2204Z in Inpatient file<br>OR<br>Mechanical ventilation ICD-10 codes (invasive and noninvasive): 5A1935Z, 5A1945Z, 5A1955Z, 5A09357, 5A09358, 5A0935Z, 5A09457, 5A09458, 5A0945Z, 5A09557, 5A09558, 5A0955Z in Inpatient file
Feeding tube placement	ICD-10 codes: 0DH64UZ, 0DH63UZ, 0DW04UZ, 0DW08UZ in Inpatient or Outpatient file<br>OR<br>CPT codes: 43246, 43653, 43830, 43832, 44300, 44372, 49440, 49441 in Carrier file

Abbreviations: CPT = Current Procedural Terminology, ICD = International Classification of Disease.

© 2025 Zhang JJ et al. *JAMA Health Forum*. All rights reserved, including those for text and data mining, AI training, and similar technologies.

eTable 2. Chronic Conditions Data Warehouse conditions

**Chronic conditions**
Acquired hypothyroidism
Acute myocardial infarction
Anemia
Asthma
Atrial fibrillation
Benign prostatic hyperplasia
Cancer, female/male breast
Cancer, colorectal
Cancer, endometrial
Cancer, lung
Cancer, prostate
Cataract
Chronic kidney disease
Chronic obstructive pulmonary disease and bronchiectasis
Depression
Diabetes
Glaucoma
Heart failure
Hip/pelvic fracture
Hyperlipidemia
Hypertension
Ischemic heart disease
Osteoporosis
Rheumatoid arthritis/osteoarthritis
Stroke/transient ischemic attack

© 2025 Zhang JJ et al. *JAMA Health Forum*. All rights reserved, including those for text and data mining, AI training, and similar technologies.

eTable 3. Frailty and functional impairment index

Frailty and functional impairment category	ICD-10 diagnosis codes and HCPCS codes
Abnormality of gait	R26.0, R26.1, R26.89, R26.9
Abnormal loss of weight and underweight	R63.4, R63.6
Adult failure to thrive	R62.7
Cachexia	R64
Anorexia	R63.0
Debility	R54
Difficulty in walking	R26.2
History of fall	Z91.81
Muscular wasting and disuse atrophy	M62.50
Muscle weakness	M62.81
Pressure ulcer	L89.XX
Senility without mention of psychosis	R41.81
Malaise and fatigue	R53.1, R53.81, R53.83
Hospital beds and associated supplies	E0250-E0373
Wheelchairs, components, and accessories	E0950-E1298, E2201-E2294, E2300-E2399, E2601-E2621, K0001-K0195, K0669
Accessories for oxygen delivery devices	E1353-E1406
Walking aids and attachments	E0100-E0159
Transportation services including ambulance	A0021-A0999

Abbreviations: HCPCS = Healthcare Common Procedure Coding System, ICD = International Classification of Disease.

© 2025 Zhang JJ et al. *JAMA Health Forum*. All rights reserved, including those for text and data mining, AI training, and similar technologies.

eTable 4. Differential changes in end-of-life care processes, outcomes, and spending for ACO beneficiaries, as compared with the control group (using the event study design)

	Differential change, percentage points [95% CI]					
**End-of-life care processes, outcomes, and spending**	**Year -2**	**Year -1**	**Year 0 (Omitted)**	**Year 1 (ACO entry)**	**Year 2**	**Year 3**
Advance care planning	-0.5 [-2.2, +1.2]	+1.1 [-0.1, +2.3]		+0.6 [-0.4, +1.7]	+0.2 [-1.1, +1.5]	+1.3 [-0.5, +3.1]
Palliative care counseling[a]	+0.4 [-1.4, +2.3]	+0.6 [-0.6, +1.8]		-0.2 [-1.2, +0.8]	-0.8 [-1.9, +0.3]	+0.3 [-1.2, +1.9]
Hospice[a]	+0.1 [-2.2, +2.4]	-0.6 [-2.2, +0.9]		-0.5 [-1.7, +0.7]	-0.6 [-1.9, +0.7]	-1.5 [-3.3, +0.3]
ED visit[b]	+0.7 [-1.2, +2.7]	+0.2 [-1.3, +1.6]		-0.5 [-1.8, +0.8]	-0.4 [-1.9, +1.1]	-0.9 [-2.9, +1.2]
Hospitalization[b]	+0.6 [-1.4, +2.6]	+0.3 [-1.2, +1.8]		-0.8 [-2.2, +0.6]	-0.2 [-1.7, +1.3]	-0.9 [-3.0, +1.2]
ICU admission[b]	+0.5 [-1.4, +2.4]	+1.6 [+0.3, +2.9][c]		+0.1 [-1.0, +1.2]	+0.5 [-0.6, +1.7]	+0.2 [-1.3, +1.7]
In-hospital death	+1.2 [-0.4, +2.8]	+0.5 [-0.6, +1.5]		-0.3 [-1.2, +0.6]	-0.3 [-1.3, +0.7]	+0.5 [-0.9, +1.8]
CPR or mechanical ventilation[b]	-1.4 [-2.7, -0.1][c]	-0.3 [-1.2, +0.5]		+0.0 [-0.8, +0.8]	-0.6 [-1.5, +0.3]	+0.4 [-0.8, +1.6]
Feeding tube placement[b]	-0.2 [-0.8, +0.4]	-0.2 [-0.6, +0.1]		+0.1 [-0.2, +0.4]	+0.0 [-0.3, +0.3]	+0.2 [-0.2, +0.6]
Total health care spending[a]	+$835 [-$988, +$2,658]	+$1,040 [-$53, +$2,133]		-$99 [-$1,016, +$818]	-$661 [-$1,658, +$337]	-$208 [-$1,897, +$1,481]

Notes. Data are of 20% random sample of Medicare beneficiaries ≥66 years with dementia who died in 2017-2020. Beneficiaries were attributed to an Accountable Care Organization (ACO) versus non-ACO. Linear probability (binary outcomes) and linear regression (spending) models were adjusted for beneficiary characteristics (age, sex, race and ethnicity, long-term nursing home resident status, median zip-code level annual household income, dual Medicare-Medicaid coverage, coexisting conditions, frailty, and Hierarchical Condition Category [HCC] risk score), included fixed effects for each unique ACO, Hospital Referral Region (HRR), year of death, and interaction of HRR and year of death, and clustered at the ACO level for beneficiaries attributed to ACO and HRR level for non-ACO beneficiaries. "Time zero" was defined as the year prior to ACO entry. *P* values were adjusted with the Holm-Bonferroni method to account for multiple comparisons (adjusted $P < 0.05$ is statistically significant). Abbreviations: CPR = cardiopulmonary resuscitation, ED = emergency department, ICU = intensive care unit. [a]In last 6 months of life. [b]In last 30 days of life. [c]Adjusted *P* not statistically significant.

© 2025 Zhang JJ et al. *JAMA Health Forum*. All rights reserved, including those for text and data mining, AI training, and similar technologies.

eTable 5. Differential changes in end-of-life health care spending for ACO beneficiaries, as compared with the control group (difference-in-differences estimates)

Health care spending in last 6 months of life, $	Baseline mean among ACO beneficiaries[a]	Difference-in-differences estimate [95% CI]	*P* value	Adjusted *P* value
Carrier (physician services)	5,873	-140 [-269, -12]	0.03	0.20
Inpatient	18,372	-579 [-1,129, -29]	0.04	0.20
Outpatient	2,986	-42 [-158, +73]	0.47	>0.99
Home health	2,022	+49 [-12, +109]	0.11	0.46
Skilled nursing facility	6,411	+45 [-251, +342]	0.76	0.76
Hospice	6,052	+36 [-158, +229]	0.72	>0.99

Notes. Data are of 20% random sample of Medicare beneficiaries ≥66 years with dementia who died in 2017-2020. Beneficiaries were attributed to an Accountable Care Organization (ACO) versus non-ACO. Linear regression models were adjusted for beneficiary characteristics (age, sex, race and ethnicity, long-term nursing home resident status, median zip-code level annual household income, dual Medicare-Medicaid coverage, coexisting conditions, frailty, and Hierarchical Condition Category [HCC] risk score), included fixed effects for each unique ACO, Hospital Referral Region (HRR), year of death, and interaction of HRR and year of death, and clustered at the ACO level for beneficiaries attributed to ACO and HRR level for non-ACO beneficiaries. *P* values were adjusted with the Holm-Bonferroni method to account for multiple comparisons (adjusted $P < 0.05$ is statistically significant).
[a]Calculated as the unadjusted mean prior to ACO entry among beneficiaries attributed to ACO.

© 2025 Zhang JJ et al. *JAMA Health Forum*. All rights reserved, including those for text and data mining, AI training, and similar technologies.

eTable 6. Characteristics of the study population at baseline using inverse probability weighting

Characteristics	Overall (N=321,798)	ACO (n=161,089)	Non-ACO (n=160,709)
Age, mean, years	84.9	84.9	85.0
Female, %	58.3	58.3	58.2
Race and ethnicity, %			
Non-Hispanic White	83.0	82.9	83.2
Non-Hispanic Black	7.9	7.9	7.9
Hispanic	5.1	5.2	5.1
Other	3.9	4.0	3.9
Median zip-code level annual household income, mean, $	$66,254	$66,159	$66,348
Dual Medicare-Medicaid coverage, %	13.5	13.6	13.4
Long-term nursing home resident, %	8.7	8.7	8.8
Selected coexisting conditions, %			
Chronic kidney disease	55.6	55.6	55.6
Heart failure	46.1	46.2	46.0
Diabetes	40.0	39.9	40.1
Chronic obstructive pulmonary disease	28.9	29.0	28.8
Cancer	15.4	15.3	15.4
Count of coexisting conditions, mean	6.5	6.5	6.5
Frail, %	72.8	72.9	72.8
HCC risk score, mean	3.2	3.2	3.2
Year of death, %			
2017	24.5	24.5	24.6
2018	24.5	24.5	24.5
2019	23.8	23.8	23.8
2020	27.1	27.2	27.1

Notes. Data are of 20% random sample of Medicare beneficiaries ≥66 years with dementia who died in 2017-2020. Beneficiaries were attributed to an Accountable Care Organization (ACO) versus non-ACO. Characteristics were measured in the baseline year prior to year of death except for long-term nursing home resident status which was determined based on the most recent Minimum Data Set (MDS) assessment within 90 days before death. Indicators for coexisting conditions included 25 non-dementia-related Chronic Conditions Data Warehouse (CCW) conditions, and count excluded cataracts and glaucoma. Frailty was defined as an indicator based on ≥2 categories of claims-based surrogates of frailty. For inverse probability weighting, we fit a logistic model predicting attribution to ACO status as a function of observed covariates above (excluding count of coexisting conditions) to generate propensity scores for ACO and non-ACO. Abbreviations: HCC = Hierarchical Condition Category.

© 2025 Zhang JJ et al. *JAMA Health Forum*. All rights reserved, including those for text and data mining, AI training, and similar technologies.

eTable 7. Differential changes in end-of-life care processes, outcomes, and spending for ACO beneficiaries, as compared with the control group (difference-in-differences estimates with inverse probability weighting)

End-of-life care processes, outcomes, and spending	Baseline mean among ACO beneficiaries[a]	Difference-in-differences estimate, percentage points [95% CI]	*P* value	Adjusted *P* value
Advance care planning	15.2%	-0.0 [-1.1, +1.1]	0.97	>0.99
Palliative care counseling[b]	17.8%	-0.7 [-1.6, +0.2]	0.11	>0.99
Hospice[b]	65.5%	-0.2 [-1.3, +0.9]	0.67	>0.99
ED visit[c]	53.1%	-0.0 [-1.3, +1.2]	0.95	>0.99
Hospitalization[c]	45.2%	-0.3 [-1.5, +1.0]	0.70	>0.99
ICU admission[c]	22.7%	-0.0 [-1.0, +1.0]	0.98	0.98
In-hospital death	14.9%	-0.4 [-1.3, +0.5]	0.35	>0.99
CPR or mechanical ventilation[c]	10.4%	-0.0 [-0.8, +0.7]	0.93	>0.99
Feeding tube placement[c]	1.3%	+0.0 [-0.3, +0.3]	0.86	>0.99
Total health care spending[b]	$41,716	-$94 [-$1,064, +876]	0.85	>0.99

Notes. Data are of 20% random sample of Medicare beneficiaries ≥66 years with dementia who died in 2017-2020. Beneficiaries were attributed to an Accountable Care Organization (ACO) versus non-ACO. Linear probability (binary outcomes) and linear regression (spending) models used inverse probability weighting and were adjusted for beneficiary characteristics (age, sex, race and ethnicity, long-term nursing home resident status, median zip-code level annual household income, dual Medicare-Medicaid coverage, coexisting conditions, frailty, and Hierarchical Condition Category [HCC] risk score), included fixed effects for each unique ACO, Hospital Referral Region (HRR), year of death, and interaction of HRR and year of death, and clustered at the ACO level for beneficiaries attributed to ACO and HRR level for non-ACO beneficiaries. *P* values were adjusted with the Holm-Bonferroni method to account for multiple comparisons (adjusted *P* <0.05 is statistically significant). Abbreviations: CPR = cardiopulmonary resuscitation, ED = emergency department, ICU = intensive care unit. [a]Calculated as the unadjusted mean prior to ACO entry among beneficiaries attributed to ACO. [b]In last 6 months of life. [c]In last 30 days of life.

© 2025 Zhang JJ et al. *JAMA Health Forum*. All rights reserved, including those for text and data mining, AI training, and similar technologies.
