# Supplementary material for: End-of-Life Care and Health Care Spending for Medicare Beneficiaries With Dementia in Accountable Care Organizations
Source: JAMA Health Forum. 2025 May 9;6(5):e250731. doi: 10.1001/jamahealthforum.2025.0731 (PMC12065038; doi:10.1001/jamahealthforum.2025.0731)
Supplement: Supplement 2. — Data Sharing Statement [file jamahealthforum-e250731-s002.pdf]

Data Sharing Statement

Zhang. End-of-Life Care and Health Care Spending for Medicare Beneficiaries With Dementia in Accountable Care Organizations. *JAMA Health Forum*. Published May 09, 2025. doi:10.1001/jamahealthforum.2025.0731

Data
Data available: No

Additional Information
Explanation for why data not available: Given our Medicare data contains (encrypted) individual identifiers of Medicare beneficiaries, the Centers for Medicare & Medicaid Services (CMS) prohibits us from sharing the data with other researchers or entities. Medicare data can be purchased independently from CMS by any researcher with a valid research proposal.
